# Geographical Distribution of *MTHFR* C677T, A1298C and *MTRR* A66G Gene Polymorphisms in China: Findings from 15357 Adults of Han Nationality

**DOI:** 10.1371/journal.pone.0057917

**Published:** 2013-03-05

**Authors:** Boyi Yang, Yuyan Liu, Yongfang Li, Shujun Fan, Xueyuan Zhi, Xiangxiang Lu, Da Wang, Quanmei Zheng, Yinuo Wang, Yanxun Wang, Guifan Sun

**Affiliations:** 1 Department of Occupational and Environmental Health, College of Public Health, China Medical University, Shenyang, China; 2 Shanghai Institute of Targeted Therapy and Molecular Medicine, Shanghai, China; Tabriz University of Medical Sciences, Islamic Republic of Iran

## Abstract

**Background:**

Methylenetetrahydrofolate reductase (*MTHFR*) C677T, A1298C and methionine synthase reductase (*MTRR*) A66G polymorphisms are important genetic determinants for homocysteine (Hcy) levels, and are associated with several disorders. These polymorphisms are heterogeneously distributed worldwide. Our objective was to explore the geographical distributions of these polymorphisms in China.

**Methodologies:**

15357 healthy adults were recruited from 10 regions. Buccal samples were collected and genomic DNA was isolated. Genotyping was performed using the fluorogenic 5′-nuclease assay.

**Principal Findings:**

The prevalence of the three polymorphisms among different populations from China varied significantly and showed apparent geographical gradients. For *MTHFR* C677T, the frequencies of the 677T allele and the 677TT genotype were significantly higher among northern populations and ranged from the lowest values (24.0% and 6.4%, respectively) in Hainan (southern) to the highest values (63.1% and 40.8%, respectively) in Shandong (northern). For *MTHFR* A1298C, the 1298C allele and the 1298CC genotype frequencies were significantly higher among southern populations and increased from low values (13.1% and 1.4%, respectively) in Shandong to high values (25.7% and 6.7%, respectively) in Hainan. For A66G, the 66G allele and the 66GG genotype frequencies increased from lower values (23.7% and 5.4%, respectively) in Shandong to higher values (29.2% and 8.6%, respectively) in Hainan. The overall frequency of the 677T allele, 677TT genotype, 1298C allele, 1298CC genotype, 66G allele and 66GG genotype in the Chinese Han population was 45.2%, 23.2%, 18.6%, 3.9%, 25.7%, and 6.6%, respectively. No gender differences were found in the prevalence of both the *MTHFR* C677T and *MTRR* A66G polymorphisms.

**Conclusions:**

This study indicates that there are marked geographical variations in the prevalence of the three polymorphisms among Chinese Han populations. Our baseline data may be useful for future researches in related fields.

## Introduction

Hyperhomocysteinemia (HHcy) is a medical condition characterized by high concentrations of plasma homocysteine (Hcy) and it has been associated with increased risk for many disorders, including vascular and neurodegenerative diseases, pregnancy complications, male infertility, birth defects, diabetes, renal diseases, osteoporosis, neuropsychiatric disorders and cancers [1]–[3]. Plasma levels of Hcy are influenced by both environmental and genetic factors. The main environmental factors include vitamins folate, B_12_, B_6_, and B_2_ [4]–[7]. The major genetic factors are polymorphisms in genes encoding homocysteine metabolizing enzymes, such as methylenetetrahydrofolate reductase (*MTHFR*) C677T, A1298C and methionine synthase reductase (*MTRR*) A66G polymorphisms.

*MTHFR* plays a critical role in catalyzing the irreversible reduction of 5,10-methylenetrahydrofolate to 5-methyltetrahydrofolate, which serves as the methyl donor for the vitamin-B_12_-depedent remethylation of homocysteine to methionine and for the synthesis of purine, DNA, and RNA. Several polymorphisms in the *MTHFR* have been identified, however, only the C677T and A1298C polymorphisms have been expressed and confirmed to affect the enzyme activity [8], [9]. The C677T polymorphism is a C to T transition at base pair 677 resulting in an alanine to valine substitution. This polymorphism encodes for a thermolabile variant that decreases enzyme activity by 65% and increases plasma total homocysteine (tHcy) levels especially under the conditions of low dietary folate [10]. The A1298C polymorphism is an A to C transition at base pair 1298 leading to a glutamate to alanine substitution. The polymorphism results in the reduction of the enzyme activity, although to lesser extent than the C677T polymorphism [11]. Neither the homozygous nor the heterozygous state for the A1298C polymorphism is associated with increased plasma homocysteine and/or lower blood folate concentrations. However, combined heterozygosity for the A1298C and C677T polymorphisms is associated with decreased enzyme activity, elevated plasma homocysteine concentrations and reduced plasma folate levels [9].

*MTRR* is another key enzyme involved in homocysteine metabolism, which is responsible for the remethylation of Hcy to methionine via a vitamin-B_12_-dependent reaction. The *MTRR* restores methionine synthase (*MTR*) activity and is consequently a critical determinant of Hcy levels [12]. The most common polymorphism in the *MTRR* gene is the substitution of A for G at nucleotide 66 (A66G), which decreases the enzyme activity and the rate of Hcy remethylation [13].

Many epidemiological studies have indicated that the *MTHFR* C677T, A1298C and *MTRR* A66G polymorphisms are associated with increased risk for several disorders aforementioned [1], [14]–[20]. The prevalence of the three polymorphisms varies in different geographical regions and ethnic groups. For example, the *MTHFR* 677T allele frequency is often reported to be high in Europe (24.1–64.3%), and low in Africa (0–35.5%). The *MTHFR* 1298C allele frequency is approximately 20–70% in Asia, 24–46% in Europe, and 0–15% in America, although it has not been more extensively studied than the C677T [21], [22]. The highest and the lowest *MTRR* 66G allele frequencies are reported among Hispanics (28.65%) and Caucasians (54.4%), respectively [23]. Additionally, it is of interest that a north-to-south cline of increase in the *MTHFR* 677T allele frequency has been observed among Europeans and North Americans [24], [25], but a reverse trend has also been reported among Pakistanis, Indians, and Chinese minority groups [26]–[28]. The reasons for these distributional differences are still unclear and can involve both genetic and environmental factors. Nevertheless, accurate information on the prevalence of these polymorphisms may be beneficial to studies of gene-disease associations, population genetics and health impact evaluation.

Heretofore, the studies on the three polymorphisms are performed mainly among European and North American populations, and the data on the distributions of these polymorphisms among Chinese population are limited. The Han nationality is the largest among the 56 nationalities in China, making up 92% of the total population. The Han population are distributed widely from north to south of China. Due to high geographical, social and dietary diversity in China, we hypothesized a varied prevalence of these polymorphisms among the Chinese Han populations residing in different regions. In addition, due to the changing of life styles and dietary habits, like in any other developing countries the rates of Hcy-related diseases, such as stroke, coronary heart disease, hypertension, are markedly increasing in China [29]. It is therefore essential to investigate the heterogeneity in the prevalence of these polymorphisms among the Chinese Han population and compare the genetic markers with other published data of different populations around the world. In this study, we showed the prevalence of the *MTHFR* C677T, A1298C and *MTRR* A66G polymorphisms among 15357 Chinese Han adults from 10 geographical regions.

## Materials and Methods

### Ethics Statement

The study was conducted in accordance with the principles stipulated by the Declaration of Helsinki and all procedures were approved by the ethics review committee of the China Medical University. All specimens and survey data were obtained with written informed consents from all participants prior to study entry and subsequently anonymised.

### Study Population

**Figure 1:**
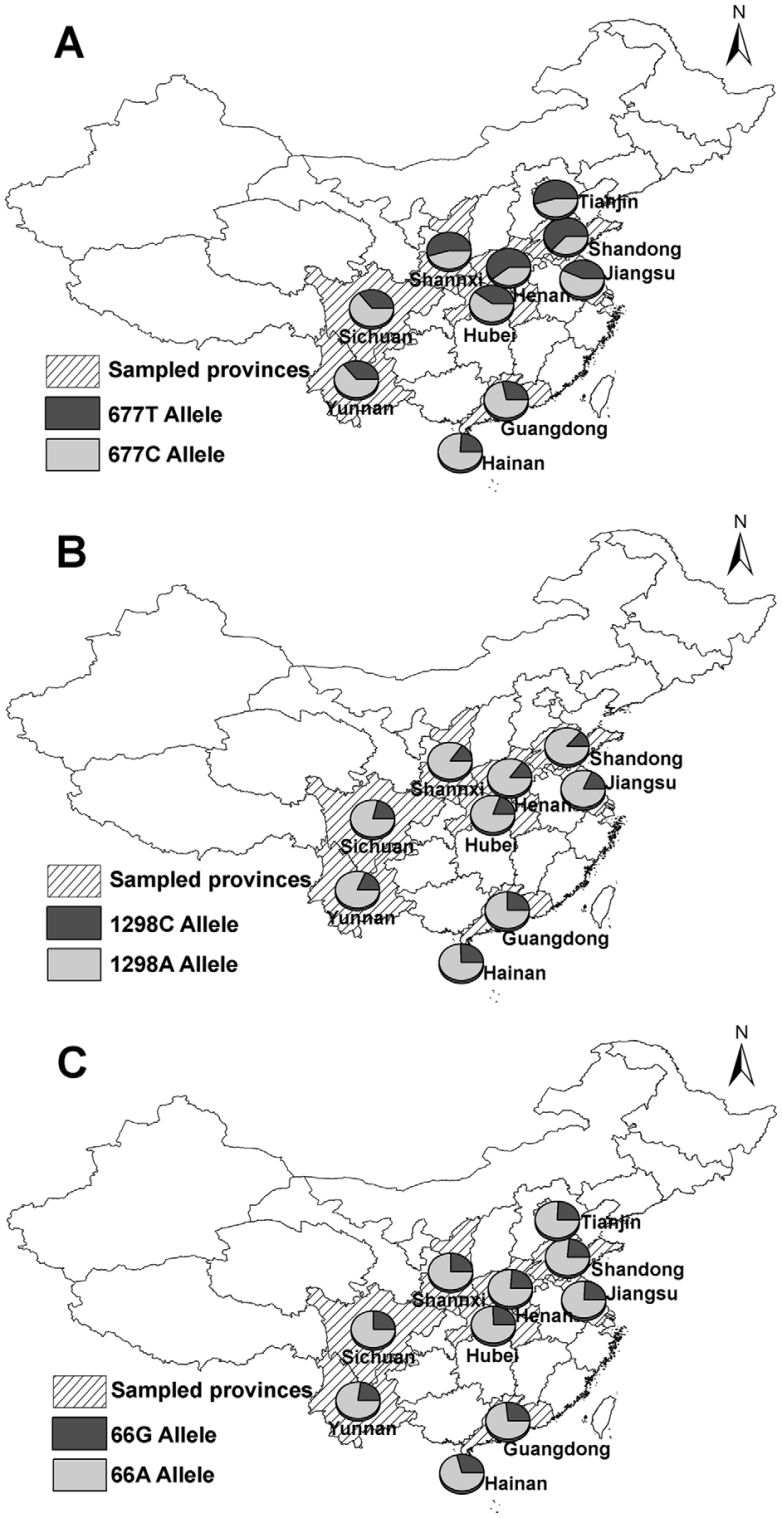
Map of China showing the distributions of the three polymorphisms in different geographical regions. Figure 1A, 1B, and 1C show the distributions of the *MTHFR* C677T, A1298C and *MTRR* A66G polymorphisms, respectively. Circles indicate the locations of the different populations in this study.

Between October 2008 and February 2011, a total of 13473 unrelated, apparently healthy women aged from 19 to 45 years (mean age, 27±4 years) who came to local maternal and children’s hospital for pre-pregnancy care, were recruited in the study. The hospitals were located in Shandong, Henan, Shannxi, Jiangsu, Hubei, Sichuan, Yunnan, Guangdong, and Hainan provinces, respectively. In addition, 1884 healthy subjects (952 males and 932 females), aged from 18 to 47 years (mean age, 26±5 years), were collected from Dagang Oil Field General Hospital in Tianjin municipality to explore whether the prevalence of the polymorphisms is different between males and females. Figure 1 shows the location of each population in this study.

The 10 populations involved in our study were divided into two major groups, the northern and the southern, according to the divide represented by the Yangtze River. The northern group included Shandong (n = 1051), Henan (n = 2661), Tianjin (n = 932), and Shannxi (n = 3090) populations, and the southern group included Jiangsu (n = 477), Hubei (n = 475), Sichuan (n = 2108), Yunnan (n = 106), Guangdong (n = 125), and Hainan (n = 3016) populations.

After obtaining due informed written consent, buccal smears were collected from consenting individuals, dried at room temperature for 1 h and then sent to the laboratory after encoding each sample.

### Genotyping

Upon arrival at laboratory, buccal cells were centrifuged and genomic DNA was extracted using the QIAamp DNA Mini Kit (Qiagen, Valencia, CA). The *MTHFR* C677T, A1298C and *MTRR* A66G genotypes were determined using the fluorogenic 5′-nuclease assay (Taqman Assay; Applied Biosystems, Foster City, CA). The assays were performed using the Taqman PCR Core Reagent Kit (Applied Biosystems, Foster City, CA) according to manufacture’s instructions. The PCR primers used in the assay and probes (Taqman MGB Probes; Applied Biosystems, Foster City, CA) which were labeled with dyes (FAM or VIC) at the 5′ end, were as follows: for *MTHFR* C677T, forward primer 5′-GAAAAGCTGCGTGATGATG-3′, reverse primer 5′-TTGAAGGAGAAGGTGTC-3′, probe 1 (VIC-dye labeled) AATCGGCTCCCGC, probe 2 (FAM-dye labeled) AATCGACTCCCGC; for *MTHFR* A1298C, forward primer 5′-AAGAACGAAGACTTCAAA-3′, reverse primer 5′- TGGGGGGAGGAGCTGAC-3′, probe 1 (VIC-dye labeled) ACACTTGCTTCACT, probe 2 (FAM-dye labeled) ACACTTTCTTCACT; for *MTRR* A66G, forward primer 5′-AGGCAAAGGCCATCGCA-3′, reverse primer 5′-ATCCATGTACCACAGCTT-3′, probe 1 (VIC-dye labeled) AAGAAATATGTGAG; probe 2 (FAM-dye labeled) AAGAAATGTGTGAG. PCR amplification using approximately 5 ng/sample of genomic DNA was done in a thermal cycler (GeneAmp PCR system 9700; Applied Biosystems, Foster City, CA). Amplifications consisted of an initial step of 95°C for 10 minutes followed by 20 cycles of 92°C for 15 seconds and 60°C for 1 minute. PCR and post-PCR fluorescence analysis were carried out on the Applied Biosystems 7900HT Sequence Detection System (Applied Biosystems, Foster City, CA), and the results were analyzed using the Applied Biosystems Sequence Detection Systems (SDS 2.2.1) software (Applied Biosystems, Foster City, CA).

### Statistical Analysis

After the genotype of each individual was obtained, allele and genotype frequencies for the *MTHFR* C677T, A1298C and *MTRR* A66G polymorphisms were calculated by direct counting. The confidence intervals of allele and genotype frequencies were computed using the normal approximation and correction for continuity. The χ^2^ analysis was performed to test Hardy-Weinberg equilibrium among each population under surveyed and compare the differences among the 10 populations with respect to allele and genotype frequencies. The differences in the prevalence of the *MTHFR* C677T and *MTRR* A66G polymorphisms between males and females were also examined by χ^2^ analysis. The comparison of allelic and genotypic frequencies between the northern and southern groups was examined using Kruskal-Wallis test. A *P* value below 0.05 was taken as statistically significant. All analyses were performed using SAS version 9.2 (SAS Institute, Cary, CN).

## Results

### Geographical Distribution of MTHFR C677T Allele and Genotype

The allele and genotype frequencies of the *MTHFR* C677T polymorphism by geographical region are given in Table 1 and Figure 1A. Our data showed a marked heterogeneity in the prevalence of the 677T allele (*P*<0.0001) and the 677TT genotype (*P*<0.0001) among the 10 populations. The prevalence of the 677T allele and the 677TT genotype seemed to have an apparent increasing cline from the south to the north. For example, the frequencies of the 677T allele and the 677 TT genotype increased from low values (24.0% and 6.4%, respectively) in Hainan, to intermediate values (28.5–43.5% and 8.3–19.7%, respectively) in Guangdong, Yunnan, Sichuan, Hubei, and Jiangsu, to higher values (54.3–60.2% and 30.4–37.0%, respectively) in Shannxi, Tianjin, and Henan, peaking in Shandong (63.1% and 40.8%, respectively). The frequencies of the 677T allele and the 677TT genotype were significantly higher in the northern than in the southern populations (*P = *0.0105 and *P = *0.0103, respectively). When taken all populations together, the frequencies of the 677TT genotype and the 677T allele in the Chinese Han population were 23.2% and 45.2%, respectively.

**Table 1:** Distribution of *MTHFR* C677T polymorphism among populations from 10 regions in China^a^.

Study areaand group	Number of subjects	Genotype (No.)			T allele frequency (%)^b^		TT genotype frequency (%)^c^	
		CC	CT	TT	Frequency	95% CI	Frequency	95% CI
**Northern** * #								
Shandong	1052	154	469	429	63.1	(60.9–65.1)	40.8	(37.8–42.9)
Henan	2661	441	1236	984	60.2	(58.9–61.5)	37.0	(35.1–38.8)
Tianjin	932	199	450	283	54.5	(53.1–55.6)	30.4	(28.7–32.0)
Shannxi	3090	670	1482	938	54.3	(52.2–56.8)	30.4	(27.4–33.4)
**Southern**								
Jiangsu	477	156	227	94	43.5	(40.3–46.7)	19.7	(16.2–23.6)
Hubei	475	172	223	80	40.3	(37.2–43.5)	16.8	(13.6–20.5)
Sichuan	2108	882	936	290	36.0	(34.5–37.4)	13.8	(12.3–15.3)
Yunnan	124	53	52	19	36.3	(30.3–42.6)	15.3	(9.5–22.9)
Guangdong	470	241	190	39	28.5	(25.6–31.5)	8.3	(6.0–11.2)
Hainan	3016	1763	1061	192	24.0	(24.6–26.8)	6.4	(5.5–7.3)
**Total**	14405	4731	6326	3348	45.2	(44.6–45.8)	23.2	(22.6–23.9)

Abbreviation: *MTHFR*, methylenetetrahydrofolate reductase; CC, “wild-type” homozygosity; CT, heterozygosity; TT, mutant homozygosity; CI, confidence interval.

a The 10 regions include 9 provinces (Shandong, Henan, Shannxi, Jiangsu, Hubei, Yunnan, Guangdong, and Hainan) and 1 municipality (Tianjin).

b The 677T allele frequencies were significantly different among the 10 populations (χ^2^ = 2166.61, *P*<0.0001).

c The 677TT genotype frequencies were significantly different among the 10 populations (χ^2^ = 1242.20, *P*<0.0001).

* The 677T allele frequencies were significantly different from the southern populations (χ^2^ = 6.55, *P = *0.0105).

# The 677TT genotype frequencies were significantly different from the southern populations (χ^2^ = 6.59, *P = *0.0103).

### Geographical Distribution of MTHFR A1298C Allele and Genotype

Table 2 and Figure 1B show the prevalence of the *MTHFR* A1298C polymorphism by geographical region. The frequencies of the 1298CC genotype varied significantly among different populations (*P*<0.0001), as well as the frequencies of the 1298C allele (*P*<0.0001). Geographically, unlike C677T, the distribution of the A1298C polymorphisms showed a reverse trend: increasing from the north to the south. For example, the 1298C allele and the 1298CC genotype frequencies were low in Shandong (13.1% and 1.4%, respectively), intermediate in Jiangsu, Hubei and Sichuan (17.8–20.7% and 3.7–5.0%, respectively), and high in Hainan (25.7% and 6.7%, respectively). The 1298C allele and the 1298CC genotype frequencies were significantly higher in the southern than in the northern populations (*P = *0.0201 and *P = *0.0389, respectively). In the total sample, the frequencies of the 1298 CC genotype and the 1298C allele were 3.9% and 18.4%, respectively.

**Table 2:** Distribution of *MTHFR* A1298C polymorphism among populations from 9 provinces in China.

Study areaand group	Number of subjects	Genotype (No.)			C allele frequency (%)^a^		CC genotype frequency (%)^b^	
		AA	AC	CC	Frequency	95% CI	Frequency	95% CI
**Northern** * #								
Shandong	1052	791	246	15	13.1	(11.7–14.6)	1.4	(0.8–2.3)
Henan	2661	1970	625	66	14.2	(13.3–15.2)	2.5	(1.9–3.1)
Shannxi	3090	2243	778	69	14.8	(13.9–15.7)	2.2	(1.7–2.8)
**Southern**								
Jiangsu	477	325	134	18	17.8	(15.4–20.4)	3.7	(2.3–6.0)
Hubei	475	318	134	23	18.9	(16.5–21.6)	4.8	(3.1–7.2)
Sichuan	2108	1340	663	105	20.7	(19.5–22.0)	5.0	(4.1–5.9)
Yunnan	124	82	39	3	18.1	(13.6–23.5)	2.4	(0.5–6.9)
Guangdong	470	262	181	27	25.0	(22.3–27.9)	5.7	(3.8–8.2)
Hainan	3016	1669	1144	203	25.7	(24.6–26.8)	6.7	(5.9–7.7)
**Total**	13473	9000	3944	529	18.6	(18.1–19.0)	3.9	(3.6–4.3)

Abbreviation: *MTHFR*, methylenetetrahydrofolate reductase; AA, “wild-type” homozygosity; AC, heterozygosity; CC, mutant homozygosity; CI, confidence interval.

a The 1298C allele frequencies were significantly different among the 9 populations (χ^2^ = 406.85, *P*<0.0001).

b The 1298CC genotype frequencies were significantly different among the 9 populations (χ^2^ = 130.73, *P*<0.0001).

* The 1298C allele frequencies were significantly different from the southern populations (χ^2^ = 5.40, *P = *0.0201).

# The 1298 CC genotype frequencies were significantly different from the southern populations (χ^2^ = 4.27, *P = *0.0389).

### Geographical Distribution of *MTRR* A66G Allele and Genotype

The prevalence of the *MTRR* A66G polymorphism is presented in Table 3 and Figure 1C. The frequencies of the 66G allele and the 66GG genotype differed significantly among the 10 populations (*P*<0.0001 and *P = *0.0003, respectively). The prevalence of the *MTRR* A66G polymorphism increased in a southerly direction. For example, the 66G allele frequency was lowest (23.7%) in Shandong and Henan, intermediate (25.2–26.8%) in Tianjin, Shannxi, Jiangsu, Hubei, Sichuan and Guangdong, and highest (29.2%) in Hainan. The frequency of the 66GG genotype showed similar variability (from 5.4% in Shandong to 8.6% in Hainan). The differences in the 677T allele and the 677TT genotype frequencies between the northern and southern groups did not reach statistical significance (*P = *0.2381 and *P = *0.2850, respectively). The mean frequencies of the 66G allele and the 66GG genotype in the Chinese Han population were 25.7% and 6.6%, respectively.

**Table 3:** Distribution of *MTRR* A66G polymorphism among populations from 10 regions in China^a^.

Study areaand group	Number of subjects	Genotype (No.)			G allele frequency (%)^b^		GG genotype frequency (%)^c^	
		AA	AG	GG	Frequency	95% CI	Frequency	95% CI
**Northern** *								
Shandong	1052	611	384	57	23.7	(21.9–25.5)	5.4	(4.1–7.0)
Henan	2661	1554	951	156	23.7	(22.6–24.9)	5.9	(4.6–6.4)
Tianjin	932	524	341	67	25.5	(23.5–27.5)	7.2	(5.6–9.0)
Shannxi^d^	3090	1706	1208	176	25.2	(24.2–26.3)	5.7	(4.9–6.6)
**Southern**								
Jiangsu	477	263	185	29	25.5	(22.7–28.4)	6.1	(4.1–8.6)
Hubei	475	261	185	29	25.6	(22.8–28.5)	6.1	(4.1–8.7)
Sichuan	2108	1188	782	138	25.1	(23.8–26.4)	6.5	(5.5–7.7)
Yunnan	124	73	46	5	22.6	(17.5–28.3)	4.0	(1.3–9.2)
Guangdong	470	254	180	36	26.8	(24.0–29.8)	7.7	(5.4–10.5)
Hainan	3016	1511	1246	259	29.2	(28.1–30.4)	8.6	(7.6–9.6)
**Total**	14405	7945	5508	952	25.7	(25.2–26.2)	6.6	(6.2–7.0)

Abbreviations: *MTRR*, methionine synthase reductase; AA, “wild-type” homozygosity; AG, heterozygosity; GG, mutant homozygosity; CI, confidence interval.

a The 10 regions include 9 provinces (Shandong, Henan, Shannxi, Jiangsu, Hubei, Yunnan, Guangdong, and Hainan) and 1 municipality (Tianjin).

b The 66G allele frequencies were significantly different among the 10 populations (χ^2^ = 58.40, *P*<0.0001).

c The 66GG genotype frequencies were significantly different among the 10 populations (χ^2^ = 31.23, *P = *0.0003).

d Deviated significantly from Hardy-Weinberg equilibrium (χ^2^ = 3.97, *P = *0.0464).

* The 66G allele and the 66GG genotype frequencies were not significantly different from the southern populations (χ^2^ = 1.39, *P = *0.2381 and χ^2^ = 1.14, *P = *0.2850, respectively).

### Prevalence of *MTHFR* C677T and *MTRR* A66G Polymorphisms by Gender

A total of 1884 samples from Tianjin municipality were collected to explore gender differences in the prevalence of the *MTHFR* C677T and *MTRR* A66G polymorphisms. Data obtained from the population are shown in Table 4 and Table 5. For *MTHFR* C677T, the 677T allele frequency in males and females was 53.7% and 54.5%, respectively. The 677TT genotype frequency in males and females was 29.3% and 30.4%, respectively. The 677T allele and the 677TT genotype frequencies were not significantly different between males and females (*P = *0.6321 and *P = *0.6157, respectively). For *MTRR* A66G, the 66G allele frequency in males and females was 23.1% and 25.5%, respectively. The 66GG genotype frequency in males and females was 6.0% and 7.2%, respectively. The 66G allele and the 66 GG genotype frequencies were not significantly different between males and females (*P = *0.0893 and *P = *0.2930, respectively).

**Table 4:** Frequencies of *MTHFR* C677T genotypes and alleles by gender^a^.

Gender	Number of subjects	*MTHFR* C677T genotype, n (%)				Allele frequency		
		CC	CT	TT	95%CI	C	T	95%CI
Males^b^	952	208 (21.9)	465 (48.8)	279 **(29.3)**	(26.4–32.3)	46.3	**53.7**	(51.5–56.0)
Females	932	199 (21.4)	450 (48.3)	283 **(30.4)**	(27.4–33.4)	45.5	**54.5**	(52.2–56.8)
Total	1884	407 (21.6)	915 (48.6)	562 **(29.8)**	(27.8–32.0)	45.9	**54.1**	(52.5–55.7)

Abbreviations: *MTHFR*, methylenetetrahydrofolate reductase; CC, “wild-type” homozygosity; CT, heterozygosity; TT, mutant homozygosity; CI, confidence interval.

a All participants were from Tianjin municipality.

b The 677T allele and the 677TT genotype frequencies were not significantly different from females (χ^2^ = 0.23, *P = *0.6321 and χ^2^ = 0.26, *P = *0.6157, respectively).

**Table 5:** Frequencies of *MTRR* A66G genotypes and alleles by gender^a^.

		*MTRR* A66G genotype, n (%)				Allele frequency		
Gender	No.	AA	AG	GG	95%CI	A	G	95%CI
Males^b^	952	569 (59.8)	326 (34.2)	57 **(6.0)**	(4.6–7.7)	76.9	**23.1**	(21.2–25.1)
Females	932	524 (56.2)	341 (36.6)	67 **(7.2)**	(5.6–9.0)	74.5	**25.5**	(23.5–27.5)
Total	1884	1093 (58.0)	667 (35.4)	124 **(6.6)**	(5.5–7.8)	75.7	**24.3**	(22.9–25.7)

Abbreviations: *MTRR*, methionine synthase reductase; AA, “wild-type” homozygosity; AG, heterozygosity; GG, homozygosity; CI, confidence interval.

a All participants were from Tianjin municipality.

b The 66G allele and the 66GG genotype frequencies were not significantly different from females (χ^2^ = 2.89, *P = *0.0893 and χ^2^ = 1.11, *P = *0.2930, respectively).

### Hardy-Weinberg Equilibrium

The observed genotype frequencies of the *MTHFR* C677T, A1298C and *MTRR* A66G polymorphisms were generally in accordance with Hardy-Weinberg equilibrium, with the exception of the *MTRR* A66G polymorphism among population from Shannxi province.

## Discussion

In this study, we examined the prevalence of the C677T and A1298C polymorphisms in the *MTHFR* gene and the A66G polymorphism in the *MTRR* gene among a large sample of Chinese adults. Our study subjects were located widely from north to south of China. The frequencies of the *MTHFR* C677T and A1298C polymorphisms differed significantly between the northern and southern populations.

Our data showed that the prevalence of the *MTHFR* 677T allele varied between 24.0% and 63.1%. The 677T allele frequencies among subjects from four northern China regions (54.3% in Shanxi, 54.5% in Tianjin, 60.2% in Henan, and 63.1% in Shandong) were much higher than those reported previously for Chinese Han populations (22.2–45.0%) [24], [25], [28], [30]. In addition to these geographical variations, it was previously suggested that 677T allele frequencies varied among ethnic groups. The average 677T allele frequency of our Han subjects was 45.2%, which was lower than that of the Tujia (55.0%) and Lahu (50.0%), and higher than that of the Duar (38.9–45.0%), Man (40.9%), She (40.0%), Xibo (37.6%), Hezhe (35.0%), Uygur (31.3%), Kazakh (30.3), Hui (29.8%), Kyrgyz (28.6%), Dai (10–27.2%), Shui (25.4%), and Jino (21.9%) [21], [28]. Globally, the prevalence of 677T allele ranged from 24.1% to 64.3% among Europeans, 6–64.3% among North Americans, 2–48.7% among South Americans, 0–35.5% among Africans, 8–31.5% among Siberians, and 2.9–28.6% among populations in Oceania [21]. Combined with our data, the frequencies of the 677T allele ranged from 2% to 63.1% in Asia [21].

Another important finding was that the 677T allele and the 677TT genotype frequencies steadily increased from southern to northern China. The geographical gradient presented in our study agrees with that previously reported among Pakistanis [26], Indians [27] and 12 Chinese minority groups [28]. However, our finding seems to contradict observations from Europe and North America, where a reverse trend was reported [24], [25], [31]. Considering the high 677T allele frequencies in Europe, low values in Africa, and the north-to-south cline of increase in the allele frequency across Europe and North American [21], [24], [25], [31], it has been presumed that high T allele frequencies occur in areas where folate intake is adequate and that low frequencies occur in areas where folate intake is inadequate [32], [33]. In China, folate intake is lower in the diet of northern populations than in that of southern populations because of differences in dietary habits [34], [35]. Therefore, we expected lower 677TT genotype frequencies among northern populations and higher frequencies among southern populations. However, our findings were contrary to our expectations. More recently, some investigators hypothesized that ultraviolet (UV) radiation could influence the distribution pattern of *MTHFR* C677T polymorphism via the photolysis of folate [36]. Lighter skin color and more exposoure to UV radiation may be a disadvantage for 677T allele carriers because of their adverse influence on folate status [37], [38]. However, the UV hypothesis still can not explain why the Chinese northerners, who had lighter skin color [39], less exposure to UV radiation [39] and lower dietary folate intake [34], [35] yet had higher folate deficiency [35] and higher 677T allele frequencies in comparison to the southerners. The reasons for the geographical gradient observed in our study are still unclear, and some other factors, such as evolution and migration in human’s history [40], may also be responsible. Further studies including genetic, nutritional, environmental and demographic factors affecting the polymorphism should be conducted to explain its distributional pattern in China.

The distribution of the *MTHFR* A1298C polymorphism is not as well studied as that of the C677T. Globally, 1298C allele frequency ranges from 10% to 70% in Asia, 24% to 46% in Europe, 13% to 32.2% in Africa, and 0% to 15% in America [22], [41]. The 1298C allele frequencies in our study ranged from 13.1% in Shandong to 25.7% in Hainan. Taken together, the mean frequency in our study was 18.4%, which is much lower than a previous report of three Han populations (41.7% in Xinjiang, 68.6% in Fujian, and 70.8% in Sichuan) and nine minority groups (25.0% among Kazak, 25.3% among Xibo, 28.3% among Man, 23.8% among Kyrgyz, 42.4% among Shui, 43.3% among Uygur, 44.6% among Jino, 47.8% among Dai, and 48.4% among Hui) [28]. The mean frequency of the1298CC genotype in this study was 3.9%, which is similar to that of Caucasians (4%), Hispanics (4%), and Italians (4.6%), but higher than that of Japanese (1.3%), Mexicans (2.6%), and African Americans (2.1%), and lower than that of Tamilians (10%), Swedish (10.4%), and Lebanese (23.9%) [42]. Interestingly, in contrast to the distribution of C677T, the prevalence of the 1298C allele and the 1298CC genotype showed a decreasing trend from southern to northern China.

The distribution of the *MTRR* A66G polymorphism is less understood. In this study, we examined the geographical variation in the distribution of the MTRR A66G polymorphism among Chinese adults. Significant deviation from Hardy-Weinberg equilibrium in Shannxi population indicates that selective pressure is acting upon this gene locus. Though the differences between various 66G allele and 66GG genotype frequencies were relatively small, the frequencies increased in a roughly southerly direction. To date, the data on the prevalence of the A66G polymorphism in China are limited. Previous studies of Chinese populations showed that 66G allele and 66GG genotype frequencies had ranges of 20–31% and 2–8%, respectively [15], [43]–[45], which affirmed our observations (25.7% and 6.6%, respectively). Worldwide distributions of the A66G polymorphism showed geographical and ethnic variations. Summaries of previous studies showed that average frequency of the 66G allele was 56.0% in Europe, 26.1% in sub-Saharan Africa, and 28.0% in Asian [46], [47]. Rady et al. [23] reported the allele frequencies among four ethnic populations in Texas: 28.7% among Hispanics, 34% among African Americans, 43.1% among Ashkenazi Jews, and 54.4% among Caucasians. In contrast, the frequency of the 66G allele in our study was relatively low.

The reasons for the distributional differences of these polymorphisms between our study and published reports are still unclear. These differences may derive from differences in study design or the age of the subjects studied, or from the partial ethnic mixing [33]. Additionally, it is possible that genetic drift, living environment, gene-gene and gene-environment interactions could effect the populational distribution of these polymorphisms [31], [32], [39], [48].

The relationship between such geographical and ethnic variation and the prevalence of related diseases is complicated. For example, in southern Italy and Spain, the *MTHFR* 677TT genotype frequencies are common, but the rates of neural tube defects and cardiovascular diseases are not very high [49], [50]. However, in Mexico, high 677TT genotype frequency is consistent with high rates of neural tube defects [51]. In North America, rates of neural tube defects are high among Hispanics, intermediate among non-Hispanics, and low among Africa Americans, a trend that follows the 677TT genotype frequency [24]. In China, we observed that 677TT genotype frequency decreased from north to south, as did rates of neural tube defects, coronary heart disease, subclinical atherosclerosis, folate deficiency and hypertension [52]–[55]. One possible explanation for these correlational variations is that genetic risks for diseases are influenced by many genetic or environmental factors [24]. Nevertheless, because of higher consistency between 677TT frequency and incidence of Hcy-related disorders in Mexico, North America, and China, the mutation could be considered a genetic risk factor for some disorders. Meanwhile, some specific preventive measures (e.g. folic acid fortification) should be taken in regions where the 677TT genotype is more frequent, such as northern China.

With respect to the *MTHFR* A1298C polymorphism, because of its weaker effect on enzyme activity and lack of association with elevated homocysteine levels [11], the majority of studies failed to find the association of the polymorphism alone with disorders [31], [56], [57]. However, compound heterozygosity for the C677T and A1298C polymorphisms was associated with elevated Hcy concentrations [9]. This interaction may partly explain previously observed contributions of the A1298C mutation to the development of some disorders [16], [17], [58]. Several epidemiological studies suggested that the *MTRR* A66G polymorphism was associated with many disorders, including birth defects [15], [18], [59]–[61], cardiovascular diseases [19], [62], [63], and cancers [20]. In addition, it was reported that interaction between the *MTRR* A66G and *MTHFR* C677T polymorphisms could increase the risk of neural tube defects [59]–[61] and schizophrenia [2]. The significant gene-gene interactions between the *MTHFR* C677T, A1298C, and *MTRR* A66G polymorphisms highlight the importance of multi-locus analyses in the risk prediction of multi-factorial disorders. This phenomenon also reminds us that not only the *MTHFR* C677T polymorphism, but also the *MTHFR* A1298C and *MTRR* A66G polymorphisms should warrant more attention, especially in regions where the *MTHFR* C677T polymorphism is less frequent and the *MTHFR* A1298C or *MTRR* A66G polymorphisms is more frequent, such as southern China.

In interpreting the findings of our study, several aspects can be criticized. Firstly, the data we used for evaluating geographical distributions are all from women. A gender effect may not be avoided because Rozen et al. [64] reported a decreased proportion of 677TT genotypes in female infants. However, when we purposely selected 1884 healthy subjects from Tianjin to explore gender differences, our study failed to find any significant differences in the prevalence of the *MTHFR* C677T and *MTRR* A66G polymorphisms between males and females, which is consistent with previous findings [31]. Secondly, our study included only 10 regions. Many other regions in China were not covered. Thirdly, compared with the other regions included in the study, the sample sizes from Yunnan province are small, which could have compromised our estimates. Despite these limitations, our study still has several strengths: (1) heretofore, having the largest sample sizes of all the studies that explore the prevalence of these polymorphisms in China, thus offering more representative and authoritative reference data; (2) reporting the distributions of three polymorphisms in 10 areas in China and specifically investigating the *MTRR* A66G polymorphism, which is scarcely reported; (3) ruling out possible differences between the polymorphic frequencies of males and females; (4) studying young subjects, thus eliminating possible impact of relationship of gene polymorphisms with a risk of shortened longevity.

In conclusion, our large-scale study indicates that the prevalence of the *MTHFR* C677T, A1298C and *MTRR* A66G polymorphisms varies significantly between Han populations residing in different regions of China. Our findings supplement worldwide reports on the three polymorphisms and are helpful for exploring the prevalence of genetic mutations in different populations. These baseline data can also facilitate interpreting the prevalence gradient of diseases associated with those polymorphisms among Chinese Han population. From a public health perspective, the data can help government and experts design preventive measures for specific populations, and evaluate the health care costs-benefits ratio in China, where dietary folic acid fortification has not been initiated.
